# A prospective evaluation of quality of life, psychosocial distress, and functional outcomes two years after radical cystectomy and urinary diversion in 842 German bladder cancer patients

**DOI:** 10.1007/s11764-024-01535-0

**Published:** 2024-01-30

**Authors:** Henning Bahlburg, Alina Reicherz, Moritz Reike, Peter Bach, Marius Cristian Butea-Bocu, Karl Heinrich Tully, Florian Roghmann, Joachim Noldus, Guido Müller

**Affiliations:** 1 Department of Urology, Marien Hospital Herne, Ruhr-University Bochum, Hölkeskampring 40, 44625 Herne, Germany; 2 Center for Urological Rehabilitation, Kliniken Hartenstein, Bad Wildungen, Germany

**Keywords:** Health-related quality of life, Psychosocial distress, Urinary incontinence, Erectile dysfunction, Radical cystectomy

## Abstract

**Purpose:**

This study aims to evaluate survival, health-related quality of life (HRQoL), psychosocial distress, and functional outcomes after radical cystectomy (RC) and urinary diversion for ileal neobladder (INB) or ileal conduit (IC) in a contemporary German cohort of bladder cancer patients.

**Methods:**

Patients undergoing inpatient rehabilitation after RC between April 2018 and December 2019 in one high-volume rehabilitation center were surveyed regarding HRQoL, psychosocial distress, and functional outcomes until two years after RC.

**Results:**

Eight-hundred forty-two patients (683 male, 159 female; 395 INB, 447 IC) were included. Patients with an IC suffered more often from locally advanced disease (≥ pT3; 41.4% vs. 24.1%, *p* < 0.001) and lymph node metastases (19.9% vs. 11.8%, *p* = 0.002), resulting in worse probability of survival (p < 0.001). Global HRQoL improved steadily during follow-up, but significant differences in subscales persisted between cohorts. Multivariable regression analysis identified IC, male sex, and patient age ≤ 59 years as independent predictors for persistent high psychosocial distress. Almost 42% of female patients reported severe urinary incontinence two years after RC. Most men reported severely diminished erectile function, even after nerve-sparing surgery.

**Conclusion:**

Global HRQoL two years after RC is comparable to the general German population. Female patients should be informed about worse continence rates. Conversely, men should be educated about erectile dysfunction. Aftercare should include psycho-oncologic counseling, especially for patients at risk.

**Implications for cancer survivors:**

Patients should be counseled on long-term functional outcomes and persistent psychosocial distress after RC. Closer cooperation between urologists and psycho-oncologists is needed.

**Supplementary Information:**

The online version contains supplementary material available at 10.1007/s11764-024-01535-0.

## Introduction

Radical cystectomy (RC) procedures have seen an increase of 28% in cases between 2006 and 2019 in Germany [1]. Ileal conduit (IC) and ileal neobladder (INB) are the most common types of urinary diversion [2]. RC may lead to a deprived body image, but comparisons of health-related quality of life (HRQoL) between patients with an IC and an INB have yielded conflicting results [3–5]. Overall, HRQoL was identified as a predictor of overall survival in several tumor entities [6–8].

Psychosocial distress is prevalent in many cancer patients and is usually driven by fear of disease progression, which may result in depression, anxiety, or an adaptive disorder [9–12]. Accordingly, psychosocial distress has been discussed as a “sixth vital sign” in cancer patients [13]. Constant monitoring for signs of psychosocial distress is recommended by current guidelines [14, 15].

Urinary incontinence after the creation of an INB may further influence QoL and has been linked to impaired emotional and cognitive function [16]. Continence rates in women are usually lower than in men [16, 17]. A recent study by our group identified increasing age, diabetes mellitus, and a body mass index (BMI) ≥ 30kg/m^2^ as independent negative predictors of urinary continence in the early postoperative period [18].

Erectile dysfunction after surgery is also known to negatively influence QoL and may lead to depression [19, 20]. Current guidelines recommend nerve-sparing surgery if feasible to improve both erectile function and urinary continence [21, 22].

Complications in the immediate period after RC, QoL and psychosocial distress 1 year after RC, and the influence of a successful return to work on QoL and psychosocial distress in this cohort have previously been reported [23–25]. An analysis of functional outcomes in 395 patients with an INB identified male sex as an independent predictor for the use of no pads, while nerve-sparing surgery was identified to predict both the use of a safety pad only and good erectile function one year after RC [26].

This study now aims to analyze 2-year outcomes after RC and urinary diversion in terms of QoL, psychosocial distress, and functional outcomes, while also reporting complications and survival.

## Methods

Clinical data of patients with urothelial carcinoma of the bladder who underwent RC with creation of IC or INB in various hospitals across Germany and who were treated in a specialized center for urological inpatient rehabilitation (Kliniken Hartenstein, Bad Wildungen, Germany) between April 2018 and December 2019 were prospectively collected. The study protocol was approved by an institutional research committee (research authorization number FF30/2017). QoL was assessed by the European Organisation for Research and Treatment of Cancer (EORTC) QLQ-C30 and QLQ-BLM30 questionnaires. The Questionnaire on Stress in Cancer Patients (QSC-R10) was used to examine psychosocial distress, while functional outcomes were assessed by the International Consultation on Incontinence – Short Form (ICIQ-SF), the International Index of Erectile Function (IIEF-5), and the Erection Hardness Score (EHS). Supplement 1 describes the questionnaires in detail. Patients were surveyed at the beginning (T1) and the end of IR (T2), 6 months (T3), 12 months (T4), and 24 months (T5) after RC. Normative data on HRQoL of the general German population were used for comparison [27]. Baseline characteristics included patient age, Karnofsky performance status, BMI, the existence of cardiovascular disease and/or diabetes, tumor stage, method of surgery, utilization of neoadjuvant chemotherapy, and socio-economic status [28]. “Social continence” was defined as the use of ≤ 1 pad per 24 h.

### Statistical analysis

Descriptive statistics for categorical variables included frequencies and proportions, while for continuous variables medians and interquartile ranges (IQR) or means and standard deviations (SD) were reported. Between-group comparisons (IC vs. INB) were analyzed using the Mann–Whitney U test or Chi-square test (Pearson) as appropriate. The Wilcoxon test was used to compare changes in quantitative variables, while a Chi-squared test (McNemar) was used to compare changes in proportions. Multivariable logistic regression analyses were performed to identify predictors of high psychosocial distress, death, and urinary continence two years after RC. Significance was considered at *p* < 0.05. Data were analyzed using SPSS version 29.0 (IBM, Chicago).

## Results

### Study cohort and baseline characteristics

Eight-hundred forty-two patients (683 male (81.1%), 159 female (18.9%)) who were treated in 135 primary German hospitals were prospectively enrolled in this study. INB was chosen for urinary diversion in 395 patients (46.9%), while 447 patients (53.1%) received an IC. Patients with an IC were significantly older (73 years (interquartile range IQR 67–78) vs. 64 years (IQR 58–69), *p* < 0.001), significantly more often suffered from locally advanced disease (tumor stage ≥ pT3 (American Joint Committee on Cancer /International Union Against Cancer (AJCC/UICC) TNM system); 41.4% vs. 24.1%, *p* < 0.001) and lymph node metastases (19.9% vs. 11.8%, *p* = 0.002), and more often reported low socioeconomic status (61.1% vs. 46.6%, *p* < 0.001), cardiovascular disease (72.9% vs. 55.9%, *p* < 0.001) and diabetes mellitus (16.1% vs. 10.6%, *p* = 0.021), respectively. Men were significantly more likely to receive an INB (52.3% vs. 47.7%, *p* < 0.001), while women were far more likely to receive an IC (76.1% vs 23.9%, *p* < 0.001). Further patient characteristics are shown in Table 1.

**Table 1:** Baseline characteristics of 842 patients after radical cystectomy for bladder cancer

Variable	Total	Conduit	Neobladder	p*
Patients, *n* (%)	842 (100.0)	447 (53.1)	395 (46.9)	
Age (years), Median (IQR)	68 (62–75)	73 (67–78)	64 (58–69)	** < 0.001**
≤ 59 years, *n* (%)	150 (17.8)	27 (6.0)	123 (31.1)	** < 0.001**
60–69 years, *n* (%)	307 (36.5)	125 (28.0)	182 (46.1)	** < 0.001**
≥ 70 years, *n* (%)	385 (45.7)	295 (66.0)	90 (22.8)	** < 0.001**
Sex, *n* (%)				
male	683 (81.1)	326 (72.9)	357 (90.4)	** < 0.001**
female	159 (18.9)	121 (27.1)	38 (9.6)	** < 0.001**
Karnofsky performance status (%), Median (IQR)	80 (70–80)	70 (70–80)	80 (70–80)	**0.021**
BMI (kg/m^2^), Median (IQR)	25 (23–28)	25 (23–28)	25 (23–27)	0.211
< 30, *n* (%)	743 (88.2)	390 (87.2)	353 (89.4)	0.341
≥ 30, *n* (%)	99 (11.8)	57 (12.8)	42 (11.6)	0.341
CCI ≥ 2, *n* (%)	128 (15.2)	89 (19.9)	39 (9.9)	** < 0.001**
Cardiovascular disease, *n* (%)	547 (65.0)	326 (72.9)	221 (55.9)	** < 0.001**
Diabetes mellitus, *n* (%)	114 (13.5)	72 (16.1)	42 (10.6)	**0.021**
Socioeconomic status**				
Low	399 (54.1)	232 (61.1)	167 (46.6)	** < 0.001**
Middle	265 (35.9)	123 (32.4)	142 (39.7)	**0.039**
High	74 (10.0)	25 (6.6)	49 (13.7)	**0.001**
Neoadjuvant chemotherapy, *n* (%)	83 (9.9)	37 (8.3)	46 (11.6)	0.102
Method of surgery, *n* (%)				
Robot-assisted cystectomy	93 (11.0)	46 (10.3)	47 (11.9)	0.458
Open cystectomy	749 (89.0)	401 (89.7)	348 (88.1)	0.458
Tumor stage, *n* (%)				
≤ pT2	562 (66.7)	262 (58.6)	300 (75.9)	** < 0.001**
≥ pT3	280 (33.3)	185 (41.4)	95 (24.1)	** < 0.001**
Lymph node positive, *n* (%)***	131 (16.1)	86 (19.9)	45 (11.8)	**0.002**
No. of lymph nodes removed, Median (IQR)	17 (12–25)	17 (12–26)	17 (12–24)	0.765

*IQR* = interquartile range; *BMI *= body mass index; *CCI *= Charlson comorbidity index

*Mann–Whitney-U test or Chi-square test (Pearson) as appropriate

**data available for 738 patients (conduit *n* = 380 and neobladder *n* = 358)

***data available for 815 patients (conduit *n* = 433 and neobladder *n* = 382)

**Bold font** indicates significant results

### Long-term complications and survival

Non-response rate at T5 was 33.6% (n = 283), including 152 (IC: n = 107, INB: n = 45, p < 0.001) patients who died during follow-up. Median overall survival was 354 days (IQR 225–579). Multivariable regression analysis identified IC (Odds ratio OR 1.784; 95% CI 1.100–2.895; p = 0.019), tumor stage ≥ pT3 (OR 5.022; 95% CI 3.298–7.646; p < 0.001), and lymph node metastases (OR 2.246; 95% CI 1.416–3.563; p < 0.001) as independent predictors for death during follow-up, while age, sex, diabetes mellitus, and cardiovascular disease were not identified as independent predictors for death within two years after RC (Table 2).

**Table 2:** Regression analysis to identify independent predictors of death within two years after radical cystectomy

Death within 2 years after radical cystectomy	univariable		multivariable	
	OR (95% CI)	*p*	OR (95% CI)	*p*
Age (continuous)	1.023 (1.003–1.044)	**0.025**	0.993 (0.967–1.019)	0.584
Conduit vs. neobladder	2.448 (1.676–3.575)	** < 0.001**	1.784 (1.100–2.895)	**0.019**
Female vs. male	1.567 (1.034–2.377)	**0.034**	0.939 (0.575–1.534)	0.802
Diabetes mellitus (yes vs. no)	0.831 (0.486–1.422)	0.500	0.695 (0.374–1.291)	0.249
CVD (yes vs. no)	1.253 (0.859–1.828)	0.241	1.234 (0.792–1.922)	0.353
Tumor stage ≥ pT3 (yes vs. no)	6.834 (4.644–10.056)	** < 0.001**	5.022 (3.298–7.646)	** < 0.001**
Positive nodal stage (yes vs. no)	4.213 (2.794–6.353)	** < 0.001**	2.246 (1.416–3.563)	** < 0.001**

*CVD *= cardiovascular disease; *OR* = odds ratio; *CI *= confidence interval; *QSC-R10* = questionnaire on stress in cancer patients (10 items)

**Bold font** indicates significant results

**Fig. 1:**
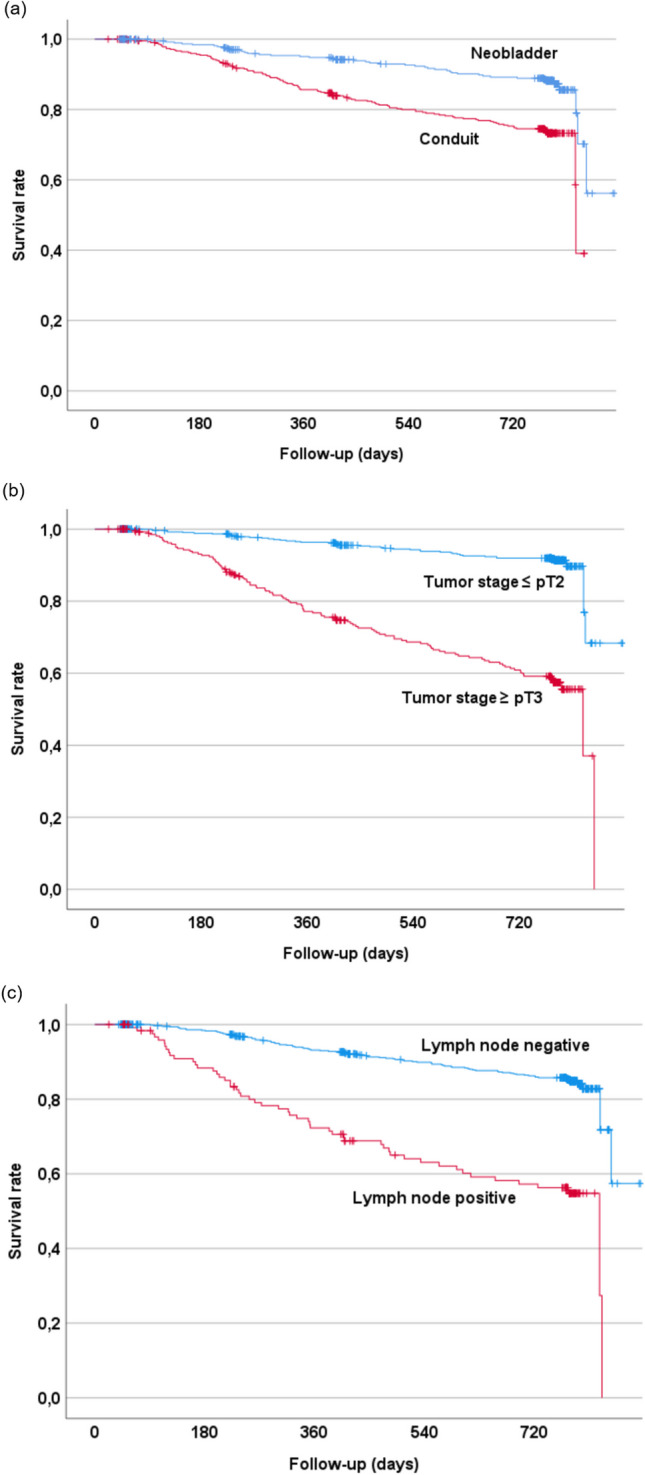
Survival rates after radical cystectomy depending on (**a**) type of urinary diversion, (**b**) tumor stage, and (**c**) nodal stage

Log-rank test showed a significantly higher probability of survival for patients with an INB (Chi-square 26.51, *p* < 0.001). Furthermore, patients with lymph node metastases and tumor stage ≥ pT3 showed worse survival rates compared to patients without lymph node metastases and tumor stage ≤ pT2 (Chi-square 74.06, *p* < 0.001 and Chi-square 133.0, *p* < 0.001), respectively (Fig. 1a-c).

Adjuvant or salvage chemotherapy was administered in 109 patients (16.1%) at T3, another 22 patients (3.8%) at T4, and an additional 18 patients (3.2%) at T5. Immunotherapy was required in 19 patients (2.8%) at T3, 12 patients (2.1%) at T4, and 16 patients (2.9%) at T5, respectively. Radiation was administered in 15 patients (2.2%) at T3, 6 patients (1.0%) at T4, and 7 patients (1.3%) at T5, respectively.

Hospital readmission was necessary for 130 patients (19.2%) at T3, 99 patients (17.2%) at T4, and 56 patients (10.0%) at T5. The most common reasons for hospital readmission were hydronephrosis (between 11.4% and 16.1% at the respective points of evaluation) and urinary tract infections (between 12.8% and 31.7% at the respective points of evaluation).

### Quality of life (EORTC-QLQ-C30 & EORTC QLQ–BLM30)

**Fig. 2:**
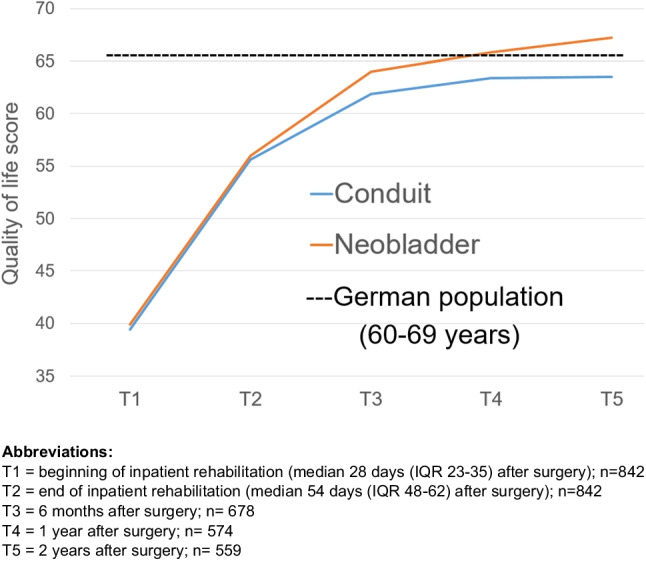
Global Health-related quality of life (EORTC QLQ-C30) after radical cystectomy

HRQoL improved steadily during follow-up. Two years after RC, global HRQoL was comparable to the general German population and did not differ between patients with IC and INB (Fig. 2).

Compared to IC patients, patients with an INB reported significantly superior physical functioning (81.0 vs. 70.6, *p* < 0.001) and role functioning (67.6 vs. 58.5, *p* < 0.001) at T5. No significant differences between types of urinary diversion were detected in the other functioning scales. At T5, patients with an INB suffered less from fatigue than patients with an IC (34.3 vs. 39.7, *p* = 0.030). While patients with an INB suffered significantly more often from diarrhea (27.7 vs. 15.4, *p* < 0.001) at T5, patients with an IC suffered significantly more often from constipation (24.2 vs. 15.7, *p* < 0.001). Financial difficulties were significantly higher in patients with an INB (19.5 vs. 11.0, *p* < 0.001). Data on all functioning scales, symptom scales, and single items can be found in Table 3a-c.

**Table 3:** QLQ-C30 functional scales (a), symptoms scales (b) and single items (c) 2 years after radical cystectomy

(a)	Total (*n* = 559) mean (SD)	Conduit (*n* = 281) mean (SD)	Neobladder (*n* = 278) mean (SD)	p*
Global HRQoL	65.3 (22.3)	63.5 (23.0)	67.2 (21.3)	0.095
Physical functioning	75.8 (22.7)	70.6 (23.9)	81.0 (20.2)	** < 0.001**
Role functioning	63.0 (31.1)	58.5 (31.1)	67.6 (30.5)	** < 0.001**
Emotional functioning	69.4 (26.6)	68.8 (26.4)	70.0 (26.9)	0.438
Cognitive functioning	79.3 (24.5)	79.8 (23.7)	78.7 (25.3)	0.782
Social functioning	66.5 (29.5)	67.9 (28.6)	65.0 (30.4)	0.339
(b)	Total (n = 559) mean (SD)	Conduit (n = 281) mean (SD)	Neobladder (n = 278) mean (SD)	p*
Fatigue	37.0 (27.3)	39.7 (28.2)	34.3 (26.2)	**0.030**
Nausea and vomiting	4.5 (13.1)	5.3 (14.7)	3.6 (11.2)	0.208
Pain	19.2 (26.7)	19.5 (27.0)	18.8 (26.5)	0.837
(c)	Total (n = 559) mean (SD)	Conduit (n = 281) mean (SD)	Neobladder (n = 278) mean (SD)	p*
Dyspnea	31.2 (32.1)	33.5 (33.9)	28.9 (30.1)	0.182
Insomnia	34.7 (34.0)	36.8 (34.9)	32.6 (33.0)	0.176
Appetite loss	12.6 (22.8)	13.2 (22.3)	11.9 (23.4)	0.222
Constipation	20.0 (28.9)	24.2 (31.1)	15.7 (25.7)	** < 0.001**
Diarrhea	21.6 (27.7)	15.4 (24.1)	27.7 (29.6)	** < 0.001**
Financial difficulties	15.2 (27.2)	11.0 (22.8)	19.5 (30.4)	** < 0.001**

*HRQoL* = health-related quality of life; *SD* = standard deviation

*Mann–Whitney-U test (ileal conduit vs. ileal neobladder)

Bold font indicates significant results

In the analysis of the results of the EORTC QLQ–BLM30 two years after RC, no differences were found between patients with an IC or an INB in term of future perspective, abdominal bloating, and self-esteem/body image (Supplement 2).

### Psychosocial distress (QSC-R10)

At T5, median QSC-R10 scores were 13 (IQR 7–21) in patients with an IC and 12 (IQR 5–20) in patients with an INB, respectively (*p* = 0.094). High psychosocial distress (QSC-R10 score ≥ 15 points) was prevalent in 42.6% (*n* = 235) of all patients, but not significantly more abundant in any form of urinary diversion (45.0% (IC) vs. 40.1% (INB), *p* = 0.252). However, patients that attended individual psychotherapy during IR also reported significantly higher psychosocial distress at T5 (median QSC-R10 score 16 points (IQR 10–24) vs. 10 points (IQR 5–18), *p* < 0.001). Additionally, patients ≤ 59 years reported higher psychosocial distress at T5 than patients ≥ 60 years (median QSC-R10 score 15 (IQR 8–22) vs. 12 (6–20), *p* = 0.022). Multivariable regression analysis identified IC (OR 1.532; 95% CI 1.048–2.239; *p* = 0.028), male sex (OR 1.669; 95% CI 1.021–2.728, *p* = 0.041), and patient age ≤ 59 years (OR 1.898; 95% CI 1.169–3.081, *p* = 0.01) as independent predictors for high psychosocial distress at T5 (Table 4).

**Table 4:** Regression analysis to identify independent predictors of high psychosocial distress (QSC-R10 score ≥ 15) two years after radical cystectomy

High psychosocial distress	univariable		multivariable	
	OR (95% CI)	p	OR (95% CI)	p
Conduit vs. Neobladder	1.218 (0.869–1.708)	0.253	1.532 (1.048–2.239)	**0.028**
male vs. female	1.518 (0.959–2.405)	0.075	1.669 (1.021–2.728)	**0.041**
Age ≤ 59 years vs. ≥ 60 years	1.552 (0.996–2.418)	0.052	1.898 (1.169–3.081)	**0.010**
Tumor stage ≥ pT3 (yes vs. no)	0.849 (0.568–1.269)	0.426	0.831 (0.539–1.281)	0.403
Positive nodal stage (yes vs. no)	1.186 (0.674–2.087)	0.553	1.303 (0.716–2.369)	0.386

*CVD* = cardiovascular disease; *OR* = odds ratio; *CI* = confidence interval; *QSC-R10* = questionnaire on stress in cancer patients (10 items)

**Bold font** indicates significant results

### Urinary continence (ICIQ-SF)

The median ICIQ score in all patients after creation of an INB was 7 (IQR 4–11) at T5. Urinary continence was reported by 13.8% (*n* = 38), while mild, moderate, and severe urinary incontinence were reported by 28.6% (*n* = 79), 29.3% (*n* = 81), and 28.3% (*n* = 78), respectively.

Male patients reported a median ICIQ score of 7 (IQR 4–11) at T5, and 13.1% (*n* = 33) reported complete urinary continence. Mild, moderate, and severe urinary incontinence were reported by 30.2% (*n* = 76), 29.8% (*n* = 75), and 27.0% (*n* = 68), respectively. At T5, median daily and nocturnal pad use in men was 1 (IQR 1–2), respectively. A “safety pad” was used by 47.2% (*n* = 119), while 35.3% (*n* = 89) did not use a pad.

Female patients reported worse urinary continence rates. The median ICIQ score in women was 10 (IQR 3–15) at T5. Complete urinary continence was reported by 20.8% (*n* = 5) at T5, while mild, moderate, and severe incontinence were reported by 12.5% (*n* = 3), 25.0% (*n* = 6), and 41.7% (n = 10), respectively. At T5, median daily and nocturnal pad use in women was 3 (IQR 1.5–4) and 1 (IQR 1–2), respectively. A “safety pad” was used by 41.7% (*n* = 10), while 20.8% (*n* = 5) did not use a pad.

While univariable regression analyses identified age ≤ 59 years (OR 1.738, 95% CI 1.014–2.978) and nerve-sparing surgery (OR 1.832, 95% CI 1.096–3.063) as predictors for the use of “no pad” at T5, multivariable regression analyses that included age, sex, BMI, diabetes mellitus, cardiovascular disease, surgical approach (open vs. robot-assisted), and nerve-sparing surgery failed to identify independent predictors for both the use of a “safety pad” or “no pad”.

Patients with “social continence” reported superior global HRQoL (70.5 vs. 60.9, *p* < 0.001) and lower PD (10 (IQR 4–18) vs. 14 (IQR 7.5–23), *p* = 0.004) than incontinent patients at T5, respectively.

### Erectile function (IIEF-5 and EHS)

At T5, only 20 men (5.3%) reported mild or no erectile dysfunction according to the IIEF-5, while 334 men (88.8%) reported complete or severe erectile dysfunction. In men with good erectile function before surgery (EHS ≥ 3), only 12.0% (*n* = 31) reported erectile function sufficient for sexual intercourse at T5. Even in men with good erectile function before RC that underwent nerve-sparing surgery, only 24.2% (*n* = 22) reported good erectile function at T5.

## Discussion

Although global HRQoL is comparable to the general German population and does not differ significantly between patients with an IC and an INB two years after RC, physical and role functioning were superior in patients with an INB. Furthermore, some previously reported differences persist in symptom scales, e.g., concerning diarrhea, constipation, and fatigue [29]. Even though we previously reported a high return to work rate two years after RC [24], financial difficulties were significantly more often reported by patients with an INB. This may be explained by their younger median age with a subsequent higher likelihood of unemployment, receiving a disability pension, or working reduced hours.

High psychosocial distress concerns 42.6% of patients two years after RC and was significantly elevated in patients that already reported high psychosocial distress during IR. Multivariable regression analyses identified IC, male sex, and age ≤ 59 years as independent predictors of high psychosocial distress at T5. These results contradict a much smaller study by Palapattu et al. of 74 patients undergoing RC that did not find an association between high psychosocial distress and age, sex, or type of urinary diversion [30]. Even though our analyses may help to identify patients at risk of persistently high psychosocial distress after RC, all patients should regularly be screened for psychosocial distress and referred for counseling if necessary. However, up to 89% of cancer patients lament insufficient psychosocial care during therapy [31].

Our study confirmed previous findings of worse urinary continence rates in women compared to men [16, 17]. Functional outcomes should be a key component when counseling female patients on urinary diversion. The importance of good functional outcomes after creation of an INB is further stressed by patients with “social continence” reporting superior HRQoL and less psychosocial distress than incontinent patients. Unfortunately, multivariable regression analysis failed to identify predictors for “social continence” in this cohort.

Even if nerve-sparing surgery is performed, most men still suffer from erectile dysfunction long-term. Potency rates after RC may reach up to 50% 10 years after surgery as reported by Schoenberg et al. [32]. However, other studies showed potency rates of 10–20% only and are more in line with our findings [33, 34]. Unfortunately, treatment of erectile dysfunction is often inadequate, as only 15% of men received sufficient treatment for erectile dysfunction after RC in an American cohort [35]. A structured penile rehabilitation with education about erectile dysfunction and treatment options could lead to improved erectile function after RC [36].

Patients with an IC have a worse probability of survival in our study. This is supported by a multivariable regression analysis, which identified IC, tumor stage ≥ pT3, and lymph node metastases as independent predictors for death within two years after RC.

Studies investigating complications after RC have mainly focused on readmission rates within 90 days of surgery [37–39]. Based on study design, we were able to identify urinary tract infections and hydronephrosis to cause most acute hospital readmissions within two years after RC.

There are several limitations to our study. Due to the study design, we could not record baseline HRQoL and psychosocial distress before RC. Secondly, our analyses do not differ between high-volume and low-volume centers. An influence of surgeon and/or center expertise and experience on functional outcomes and choice of urinary diversion, as reported by Maurice et al., therefore cannot be ruled out [40]. Furthermore, an analysis of sexual function after RC in female patients could have offered additional insights but was not carried out. Nonetheless, this study conveys important information about HRQoL, psychosocial distress, functional outcomes, survival, and long-term complications in a large, recent, representative, and prospectively compiled German cohort.

## Conclusions

Global HRQoL two years after RC is comparable to the general German population and does not differ significantly between patients with an IC and patients with an INB, although some differences persist (e.g., physical and role functioning, certain symptom scales). Men and women should be informed about potentially diminished functional outcomes. As psychosocial distress remains elevated in a substantial number of patients, psycho-oncological counseling should be made more easily accessible after RC and the creation of an IC or INB.

## Supplementary Information

Below is the link to the electronic supplementary material.

Supplementary file1 (DOCX 20 KB)

Supplementary file2 (DOCX 18 KB)

## Data Availability

Data are not publicly available but can be made available by the corresponding author upon reasonable request.

## Declarations

The study protocol was approved by the appropriate ethics committee (research authorization number FF30/2017). All patients gave their informed consent prior to their inclusion in the study.
