# Supplementary material for: A prospective evaluation of quality of life, psychosocial distress, and functional outcomes two years after radical cystectomy and urinary diversion in 842 German bladder cancer patients
Source: J Cancer Surviv. 2024 Jan 30;19(3):1102–10. doi: 10.1007/s11764-024-01535-0 (PMC12081545; doi:10.1007/s11764-024-01535-0)
Supplement: Supplementary file 1 — Supplementary file1 (DOCX 20 KB) [file 11764_2024_1535_MOESM1_ESM.docx]

**Supplement 1 - Methods**

*Quality of Life Questionnaire - EORTC QLQ-C30*

The EORTC QLQ-C30, issued by the European Organization for Research and Treatment of Cancer (EORTC) is a questionnaire specially designed to evaluate QoL in cancer patients [1]. It consists of 30 scored items and includes symptom scales such as dyspnea, loss of appetite, constipation, diarrhea and functional scales such as physical function, cognitive function, emotional function, and social function. A high score in the functional scales equates to a high QoL, while a high score in the symptom scales mirrors a higher burden of symptoms.

*Quality of Life Questionnaire – EORTC QLQ-BLM30*

The QLQ-BLM30 is an addendum to the QLQ-C30 and was developed to evaluate QoL after radical cystectomy. Disease-specific items such as micturition symptoms or problems concerning the maintenance of the urostomy as well as concerns regarding the future or a negative body image are assessed [2]. Results are interpreted following the QLQ-C30.

*Questionnaire on Stress in Cancer Patients – QSC-R10*

Patients were screened for psychosocial distress using the Questionnaire on Stress in Cancer Patients (QSC-R10), a standardized and validated 10-item self-assessment instrument [3]. The 10 items cover the most relevant psychosocial aspects of everyday life in cancer patients. Symptoms such as pain, fatigue, and weakness as well as fear of disease progression, sleep disorders, or lack of information regarding the disease and its treatment are assessed. The 10 items are answered on a scale of 0 (“not applicable”) to 5 (“very high burden”). The QSC-R10 total score is calculated by adding up the single items. A sum ≥15 mirrors a high psychosocial burden and should trigger psycho-oncological counseling.

*International Consultation on Incontinence Questionnaire – Short Form (ICIQ-SF)*

The ICIQ-SF is a validated patient-reported assessment and examines the frequency and quantity of involuntary loss of urine and its influence on QoL [4]. The questionnaire consists of three scored items, scored on a Likert-scale between 0 and 5. Zero points are equivalent to no impairment while a score of 5 points is equivalent to a very high impairment. The total sum of all three scored items allows for classifying the patients’ incontinence into three groups. A sum between 1 and 5 points is defined as mild, a score between 6 and 10 points as moderate, and a score ≥ 11 as severe incontinence [5]. Additionally, the number of pads used daily is examined. Social continence was defined as the use of a maximum of one safety pad per 24 hours.

*International Index of Erectile Function (IIEF-5)*

The IIEF-5 is a validated, patient-reported tool to measure erectile function and was developed based on the International Index of Erectile Function, containing 15 items [6]. Its five items concern erectile function, sexual desire, orgasm ability, and sexual satisfaction. Each question is scored on a Likert-scale of 1–5. 1 point equals a high impairment, and 5 points equals no impairment. The erectile function can then be classified according to the sum of all scores. A score of 5–7 points signals severe, a score between 8 and 11 moderate, a score of 12–16 weak to moderate, a score of 17–21 weak, and a score of 22–25 points no erectile dysfunction (ED).

*Erection Hardness Score (EHS)*

The EHS offers another easy tool to measure erectile function, in which the erection is scored on a 5-point Likert-scale (0–4) [7]. The result can be interpreted as follows. Zero points: the penis does not enlarge when the patient is sexually aroused. One point: the penis enlarges but does not get hard. Two points: the penis is hard but not sufficiently so for penetration. Three points: the penis is hard enough for penetration but not totally hard. Four points: the penis is hard and rigid.

[1] Aaronson NK, Ahmedzai S, Bergman B, Bullinger M, Cull A, Duez NJ, et al. The European Organization for Research and Treatment of Cancer QLQ-C30: a quality-of-life instrument for use in international clinical trials in oncology. J Natl Cancer Inst. 1993;85:365-76.

[2] Danna BJ, Metcalfe MJ, Wood EL, Shah JB. Assessing Symptom Burden in Bladder Cancer: An Overview of Bladder Cancer Specific Health-Related Quality of Life Instruments. Bladder Cancer. 2016;2:329-40.

[3] Book K, Marten-Mittag B, Henrich G, Dinkel A, Scheddel P, Sehlen S, et al. Distress screening in oncology-evaluation of the Questionnaire on Distress in Cancer Patients-short form (QSC-R10) in a German sample. Psychooncology. 2011;20:287-93.

[4] Avery K, Donovan J, Peters TJ, Shaw C, Gotoh M, Abrams P. ICIQ: a brief and robust measure for evaluating the symptoms and impact of urinary incontinence. Neurourol Urodyn. 2004;23:322-30.

[5] Klovning A, Avery K, Sandvik H, Hunskaar S. Comparison of two questionnaires for assessing the severity of urinary incontinence: The ICIQ-UI SF versus the incontinence severity index. Neurourol Urodyn. 2009;28:411-5.

[6] Rosen RC, Cappelleri JC, Smith MD, Lipsky J, Pena BM. Development and evaluation of an abridged, 5-item version of the International Index of Erectile Function (IIEF-5) as a diagnostic tool for erectile dysfunction. Int J Impot Res. 1999;11:319-26.

[7] Mulhall JP, Goldstein I, Bushmakin AG, Cappelleri JC, Hvidsten K. Validation of the erection hardness score. J Sex Med. 2007;4:1626-34.
