# Supplementary material for: A prospective evaluation of quality of life, psychosocial distress, and functional outcomes two years after radical cystectomy and urinary diversion in 842 German bladder cancer patients
Source: J Cancer Surviv. 2024 Jan 30;19(3):1102–10. doi: 10.1007/s11764-024-01535-0 (PMC12081545; doi:10.1007/s11764-024-01535-0)
Supplement: Supplementary file 2 — Supplementary file2 (DOCX 18 KB) [file 11764_2024_1535_MOESM2_ESM.docx]

**Supplement 2:** QLQ–BLM30 domains 2 years after radical cystectomy

| **Variable** | **Total**  n=559  mean (SD) | **Conduit**  n=281  mean (SD) | **Neobladder**  n=278  mean (SD) | **p*** |
| --- | --- | --- | --- | --- |
| Urinary symptoms |  |  | 34.8 (20.1) |  |
|  |  |  |  |  |
| Urostomy problems |  | 23.5 (20.3) |  |  |
|  |  |  |  |  |
| Future perspective | 42.3 (30.4) | 43.1 (30.3) | 41.6 (30.5) | 0.507 |
|  |  |  |  |  |
| Abdominal bloating / flatulence | 31.8 (27.1) | 32.0 (27.6) | 31.5 (26.6) | 0.958 |
|  |  |  |  |  |
| Self-esteem / body image | 35.2 (29.6) | 33.1 (29.6) | 37.2 (29.7) | 0.074 |

**Abbreviations:**

SD = standard deviation

*Mann-Whitney-U test (ileal conduit vs. ileal neobladder)
